# Alternatively Activated Macrophages in Types 1 and 2 Diabetes

**DOI:** 10.1155/2012/815953

**Published:** 2012-12-27

**Authors:** Arlett Espinoza-Jiménez, Alberto N. Peón, Luis I. Terrazas

**Affiliations:** Unidad de Biomedicina, Facultad de Estudios Superiores Iztacala, Universidad Nacional Autónoma de México, Avenida De los Barrios 1, Los Reyes Iztacala, 54090 Tlalnepantla, MEX, Mexico

## Abstract

Macrophages are innate immune cells derived from monocytes, which, in turn, arise from myeloid precursor cells in the bone marrow. Macrophages have many important roles in the innate and adaptive immune response, as well as in tissue homeostasis. Two major populations have been defined: The classically activated macrophages that respond to intracellular pathogens by secreting proinflammatory cytokines and reactive oxygen species and alternatively activated macrophages which are induced during Th2 responses displaying anti-inflammatory activities. Both macrophage populations are central players in diabetes, the first one triggering inflammatory responses which initiates insulitis and pancreatic *β* cell death during type 1 diabetes, whereas the second population decreases hyperglycemia, insulitis, and inflammation in the pancreas, thereby negatively regulate type 1 diabetes. Obesity is an important factor in the development of type 2 diabetes; classically activated macrophages are a dominant cell population involved in the establishment of the inflammatory profile, insulin resistance, and activation of inflammatory signals during the development and progression of this disease. In contrast, alternatively activated macrophages regulate the release of proinflammatory cytokines, attenuating adipose tissue inflammation. Here, we review the advantages and disadvantages of these two macrophage populations with regard to their roles in types 1 and 2 diabetes.

## 1. Macrophages

M*φ*s have important roles in the immune response and tissue homeostasis. The huge capacity of M*φ*s for phagocytosis renders them effective at microbial killing and the clearance of apoptotic and necrotic cells, and through their expression of MHC-II molecules and secretion of pro- and anti-inflammatory cytokines, they can also trigger CD4+ T-cell activation and differentiation into Th1, Th2, Th17, and Treg subsets [1–3]. Importantly, M*φ*s have diverse roles in the regulation of glucose and lipid metabolism, as well as in the inflammation of adipose tissue [4].

In recent years, it has been clearly demonstrated that macrophages display high plasticity depending on the microenvironment in which they are found. Two major macrophage phenotypes have been described, specifically, classically activated macrophages (CAM*φ*s) and alternatively activated macrophages (AAM*φ*s) [2]. CAM*φ*s are induced by stimulation with Th1-cell-derived IFN-*γ* and microbial products, such as bacterial lipopolysaccharide (LPS) [5], and respond to microbial infection with an enhanced phagocytic microbicidal capability through the expression of the CAMs marker, inducible nitric oxide synthase (iNOS), which catalyzes the conversion of L-arginine into ROS, such as NO. These macrophages produce several proinflammatory cytokines, such as tumor necrosis factor-alpha (TNF-*α*), interleukin-12 (IL-12), IL-1*β*, and IL-23, as well as toxic mediators, such as reactive oxygen species (ROS) and nitric oxide (NO), through the expression of inducible nitric oxide synthase (iNOS). These macrophages also have an enhanced antigen presenting ability [6].

In contrast, AAM*φ*s are induced during Th2-type responses, such as those elicited by helminthic infection and during allergic responses. The activation of these macrophages is dependent upon stimulation with IL-4/IL-13 [16] through the IL-4R*α* receptor [17] and signal transducer and activator of transcription factor 6 (STAT6) [18], as well as with several helminth antigens [19–22]. AAM*φ*s produce moderate levels of IL-10 and TGF-*β* and low or null levels of the proinflammatory cytokines secreted by CAM*φ*s. Additionally, AAM*φ*s produce urea, polyamines, and L-ornithine, due to the high expression of the enzyme arginase-1 (Arg-1), which competes for its common substrate, L-arginine, with iNOS, thereby lowering the levels of NO secretion [6, 23]. AAM*φ*s have enhanced expression of Ym-1, which induces eosinophil recruitment [24]; these cells, in turn, can potentiate the Th2 response and the alternative activation of macrophages by the secretion of the anti-inflammatory cytokines IL-4/IL-13. Further, AAM*φ*s can express high levels of PD-1 ligands (Program-Death 1), PDL-1 and PDL-2, thereby inhibiting the proliferative response of activated T-cells [25].

AAM*φ* populations have been identified as an essential part of the immune response against almost any helminth parasite, such as *Taenia crassiceps *[25, 26], *Brugia malayi *[27, 28],* Schistosoma mansoni* [29, 30], *Litomosoides sigmodontis *[31]*, Nippostrongylus brasiliensis *[32]*, Heligmosomoides polygyrus* [33],* Fasciola hepatica *[19]*, Hymenolepis diminuta *[34], and* Echinococcus granulosus* [35]. 

Of importance for this paper, helminth-induced AAM*φ*s have been linked with decreased T1D-triggering inflammation, as well as glucose tolerance induction during obesity [4], by which these macrophages may participate in inhibiting the initiation and development of both TD1 [7] and TD2 [15].

This paper focuses on the different roles that CAM*φ*s and AAM*φ*s display in both types of diabetes, emphasizing the role of AAM*φ*s as essential players in diabetes regulation. 

## 2. Diabetes Mellitus

Diabetes mellitus is a group of metabolic diseases characterized by hyperglycemia as a result of the impairment of insulin secretion, its action, or both. The chronic hyperglycemia of diabetes is associated with dysfunction and failure of various organs, such as the eyes, kidneys, heart, and blood vessels [36]. It has been estimated that the number of deaths caused by diabetes worldwide is 4.6 million per year. Thus, diabetes remains a major cause of death and is considered to be an epidemic. Diabetes mellitus is divided into two categories: type 1 diabetes (T1D) and type 2 diabetes (T2D), and at least 90% of all cases belong to the latter [37]. 

### 2.1. Type 1 Diabetes

T1D is an autoimmune disease that has increased in prevalence over the last 30 years in developed countries. It is known that more than 5.3 million people in the world have T1D, and more than 218,000 may develop the disease each year [40]. T1D is caused by the selective destruction of the insulin-producing **β** cells located in pancreatic Langerhans' islets by autoantigen-specific inflammatory T cells. Insulin, glutamic acid decarboxylase (GADA/GAA), and protein tyrosine phosphatase (IA-2AA) are the most common autoantigens involved in this process. When the majority of **β** cells are destroyed, the pancreas's ability to secrete insulin in response to blood glucose levels is impaired, resulting in the disruption of glucose homeostasis [36].

**Figure 1:**
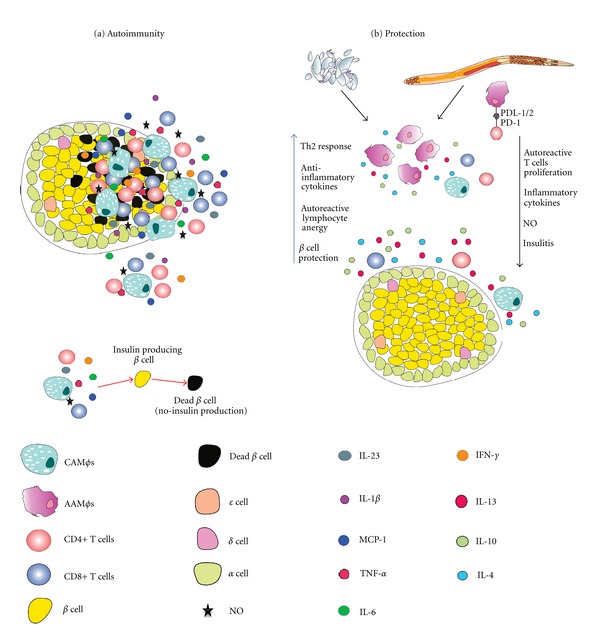
(a) In type 1 diabetes, CAM*φ*s and autoreactive T cells are the first cells that infiltrate the islets of Langerhans and release proinflammatory cytokines and NO, which induce *β* cell apoptosis or necrosis; (b) the release of anti-inflammatory cytokines, AAM*φ* induction and PD-1/PD-ligand-dependent lymphocyte anergy induction by helminths, the antigens of which have the ability to decrease NO, as well as proinflammatory cytokine, secretion, thereby reducing insulitis and *β* cell death.

CAM*φ*s and CD4+ and CD8+ autoreactive lymphocytes are the first cells that infiltrate the Langerhans islets, and the levels of cytokines, such as TNF-*α*, IL-1*β*, and IL-6, as well as NO, are increased in the pancreas during inflammation (Figure 1), where they activate different signaling pathways [38]. IL-1*β* and TNF-*α* induce the NF-*κ*B (nuclear factor *κ*B) signaling pathway, which promotes apoptosis of *β* cells by increasing the expression of FAS. TNF-*α* and IFN-*γ* act synergistically to activate the transcription factor signal transducer and activator of transcription-1 (STAT-1) signaling, thus inducing iNOS overexpression and secretion of NO and thereby promoting apoptosis of *β* cells by the p53 pathway [38, 39]. Free radicals, in turn, can induce apoptosis and necrosis of *β* cells by activating the caspase pathway and inducing excessive cell stress, respectively [39]. During this process, chemokines, such as MCP-1 (or CCL2), are also secreted; this chemokine is important in the recruitment of CAM*φ*s, inflammatory monocytes, dendritic cells, and T cells into the pancreatic islets [40, 41]. Another cytokine that has been involved in T1D is the macrophage migration inhibitory factor (MIF). MIF is associated with MCP-1, which facilitates monocyte transmigration [42]. In a mouse model with MLD-STZ, the levels of MIF were elevated in diabetic mice, and the use of MIF inhibitors reduced the inflammatory response and insulitis [43]. 

A study performed in diabetic patients showed increased numbers of monocytes, as well as higher levels of IL-1*β*, IL-6, and TNF-*α*, in the pancreas of sick patients compared with healthy people. The enhanced expression of CD80 and PDL1 in the infiltrating monocytes suggests a proinflammatory profile for these cells [44]. Several studies have attempted to verify the role of CAM*φ*s as important cells in the initiation and development of T1D. In experimental models, Martin et al. [41] demonstrated that the increased expression of CCL2 (using RIPCCL2 transgenic mice) promotes the recruitment of inflammatory monocytes to the pancreatic islets, thereby initiating inflammation and destruction of *β* cells. These data suggest that monocytes are needed for the development of diabetes. Also, the experimental depletion of CAM*φ*s in NOD mice by the intraperitoneal injection of clodronate liposomes resulted in a decrease in insulitis and inflammation [45, 46]. 

Recently, a new subpopulation of CD4+ T lymphocytes, known as Th17 cells, have been described, which are characterized by their ability to secrete high levels of IL-17, thereby promoting an inflammatory profile. The differentiation of Th17 cells is dependent upon IL-6 and transforming-growth factor-*β* (TGF-*β*) stimulation, and the presence of this subpopulation of CD4+ cells has been correlated with the onset and progression of autoimmune diseases, such as T1D [47]. IL-23 is an inflammatory cytokine involved in the expansion and commitment of Th17 cell populations, and one of its main sources is CAM*φ*s. In diabetic mice induced by streptozotocin (STZ), it has been shown that the administration of IL-23 increases IL-17, TNF-*α*, and IFN-*γ* secretion, which is associated with the onset of extremely severe T1D, implicating CAM*φ*s in the recruitment, differentiation, and expansion of pathogenic Th17 lymphocytes, contributing to *β* cell death and T1D induction [48]. Therefore, CAM*φ*s and Th17 cells, together with CD8+ cytotoxic T cells, are considered to be the main cell populations favoring the development of T1D.

However, certain pathogens (mainly viruses) can induce the development of T1D, including Rubella, enterovirus, rotavirus, cytomegalovirus, and mumps, by diverse mechanisms [49]. Several viruses may break self-tolerance by the expression of viral antigens; additionally, certain viral proteins show homology with autoantigens of *β* cells (known as molecular mimicry). Furthermore, several viruses can express superantigens, which results in an increase in the autoreactive T-cell populations, or induce the cytolysis of *β* cells, including Coxsackievirus [50] and Encephalomyocarditis (EMC) virus [51]. In the case of humans, rubella virus infection correlates with an increased incidence of T1D, and one possible mechanism of induction is molecular mimicry. Other examples are rotavirus and reovirus, which have been shown to induce lysis of *β* cells and release of autoantigens, suggesting the first mechanism of induction of T1D [49, 51]. Conversely, other pathogens may have protective roles and T1D. Epidemiological observations have pointed out an increase in the incidence and prevalence of T1D and other autoimmune diseases, mainly in developed countries, which have been correlated with a decrease in the incidence of bacterial and parasitic infections, particularly helminth infections. These observations prompted the proposal of the *hygiene hypothesis*, which states that the lack of intense infections that actively modulate the balance of the immune response toward Th2 or anti-inflammatory profiles (such as those that can be found in helminth infections) favors the induction of strong Th1 immune responses against autoantigens, thereby favoring the development of autoimmune responses [40, 52]. 

Helminths share a unique ability to exert profound regulatory effects on the immune system of their hosts by inducing strong Th2-type responses and increasing the numbers of regulatory cell populations, such as Tregs and AAM*φ*s. The results of several experiments in murine models of autoimmunity and its regulation by helminth infections support the protective role of helminth-induced Th2 responses proposed by the hygiene hypothesis [3, 53, 54]. For example, it has been shown that the infection of nonobese diabetic (NOD) mice with *Heligmosomoides polygyrus* has a protective effect in T1D, resulting in the regulation of hyperglycemia and reduced incidence of diabetes; these effects were accompanied by reduced numbers of macrophages, dendritic cells, and CD4+ and CD8+ T cells in the inflammatory infiltrate in the pancreas, as well as a reduction on *β* cell damage. Importantly, higher numbers of AAM*φ*s were found in the pancreatic and peripheral lymph nodes of NOD mice compared to noninfected mice [12]. Interestingly, in other studies, the experimental infection of mice with* Schistosoma mansoni* or their treatment with either helminth or soluble worm extracts (SWA) or soluble egg antigen (SEA) could prevent diabetes in NOD mice, with a direct relationship being observed between the lower incidence of T1D and reduced insulitis and higher numbers of AAM*φ*s [8–10]. Other regulatory cell populations, such as Treg cells, which can inhibit inflammation and suppress several autoimmune diseases, including T1D, also increased in number during *Schistosoma mansoni* infection and antigen administration [10]. Other parasites, such as *Litomosoides sigmodontis,* have also been shown to reduce T1D [13]. We have shown that previous *Taenia crassiceps* infection of diabetic mice, which were induced by multiple low doses of streptozotocin (MLD-STZ), significantly decreased the incidence of T1D, hyperglycemia, and the inflammatory infiltration of islets of Langerhans. These effects were accompanied by a significant increase in the secretion of IL-4 and the expansion of the AAM*φ*s population compared with noninfected, diabetic mice, suggesting that AAM*φ*s induced by* T. crassiceps* infection can be important in the protection against T1D [7]. In a recent study, the adoptive transfer of AAM*φ*s, which were induced *in vitro* by IL-4 and IL-13, into diabetic mice reduced kidney injury, hyperglycemia, and insulitis in the pancreas, clearly suggesting that AAM*φ*s may have a protective role against T1D [55]. In another recent study, the adoptive transfer of AAM*φ*s, which expressed PDL-2, Fc*γ*RIIb, IL-10, and TGF-*β* prevented 80% of NOD mice from developing this disease [56]. Collectively, these data suggest that AAM*φ*s may have important roles in the inhibition and prevention of T1D (Table 1 and Figure 1). 

### 2.2. Type 2 Diabetes

T2D is a metabolic disease, and its incidence has increased significantly in recent years. It is estimated that in 2000, there were approximately 171 million people with this disease, and it has been predicted that by 2030, the prevalence of T2D will increase to 366 million people [57]. T2D is characterized by a peripheral resistance to the action of insulin and a rise in insulin production by *β* cells in a process called “compensatory hyperinsulinemia” to force glucose uptake in peripheral tissues. Regardless, during T2D, there is a chronic deficiency of glucose uptake and insulin action, mainly in the liver, skeletal muscle, and adipose tissue (AT), causing hyperglycemia, hypercholesterolemia, and hyperlipidemia [58, 59]. 

AT is composed of adipocytes, preadipocytes (which are immature adipocytes that have not yet loaded any lipids), endothelial cells, leukocytes, fibroblasts, and macrophages [60]. During obesity, lipid accumulation causes a high degree of stress on adipocytes, activating them and promoting the production and subsequent release of free fatty acids (FFA), proinflammatory adipocytokines (such as leptin and resistin), and cytokines, such as IL-1*β*, IL-6, TNF-*α*, MCP-1, and MIF, as well as ROS [61–63], ensuring that in addition to its well-known capacity to store energy, AT has the capability to function as an endocrine organ. In fact, this endocrine ability of AT triggers inflammation, leading to insulin resistance and the development of T2D.

**Figure 2:**
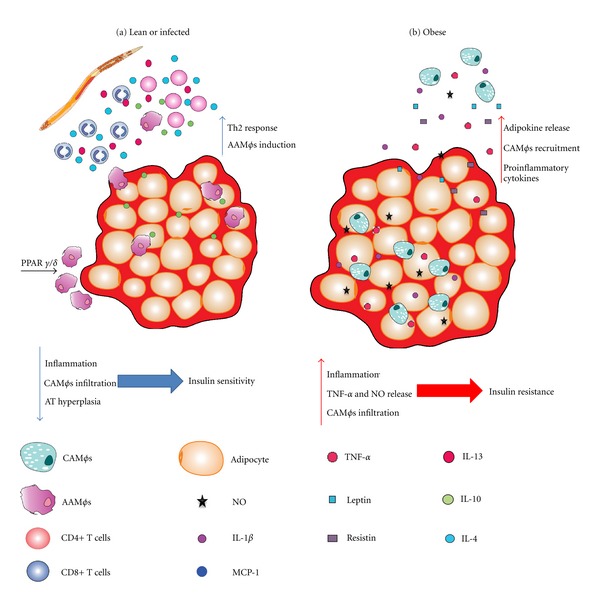
(a) Lean individuals have AAM*φ*s in their AT, which protect them from insulin resistance by secreting IL-10. An helminth infection can recruit Th2 lymphocytes, IL-4/13-secreting eosinophils and AAM*φ*s, thereby increasing protection. The natural AAM*φ* population in the lean AT is sustained by PPAR*γ*/*δ*; (b) obesity induces resistin and leptin secretion, as well as proinflammatory adipocytokines, thereby promoting CAM*φ* recruitment into the AT. CAM*φ*s in turn induce insulin resistance by secreting NO and TNF-*α*.

Several data show that macrophages are recruited into AT and classically activated due to adipocytokine secretion, contributing to the establishment of an inflammatory profile and insulin resistance in this tissue. A deficiency of MCP-1 (CCL2) or CCR2 (CCL2 receptor) in mice during obesity results in the impairment of CAM*φ* recruitment to adipose tissue, thus impeding the induction of insulin resistance by a high-fat diet (HFD) [64, 65] and suggesting an important role for CAM*φ*s in T2D initiation and development (see Figure 2). Additionally, the stressed AT secreted the adipocytokines leptin and resistin, which have been implicated in the recruitment and activation of monocytes and CAM*φ*s in adipose tissue, inducing these cells to produce higher levels of TNF-*α*, IL-12, and IL-6 [61]. Besides the production of resistin by stressed AT, stressed AT also induces the expression of MCP-1 and cellular adhesion molecules, such as V-CAM and ICAM, in adipose tissue and its vascularization [66]. Furthermore, FFA can be recognized by Toll-like receptors (TLRs) with low affinity, leading to the activation of macrophages, which release more TNF-*α* [67, 68]. TNF-*α* (one of the cytokines most abundantly secreted by CAM*φ*s) has the ability to reduce the expression of important genes in the glucose regulation process, such as the glucose transporter GLUT-4 [4]; in fact, TNF-*α* receptor knock out mice are resistant to diabetes induction [69], suggesting that the endocrine function of AT is important in the recruitment and activation of CAM*φ*s and the induction of insulin resistance. Consistent with these observations, a recent report on a model of T2D (induced with a single high dose of streptozotocin) in MIF KO mice showed that these mice had a reduced inflammatory response, such as reduced TNF-*α* production, and failed to develop T2D, demonstrating that MIF is also important in promoting the disease [70].

Secretion of IL-1*β*, TNF-*α*, and ROS by AT CAM*φ*s induces the activation of JNK and NF-*κ*B signaling in various leukocytes. NF-*κ*B is a transcription factor with an important role in the induction of inflammatory responses and the activation of CAM*φ*s, whereas JNK (c-Jun amino-terminal kinase), also known as the protein kinase activated by stress (SAPK), is activated by oxidative stress. Therefore, the activation of these signaling pathways induces the production of more IL-1*β*, TNF-*α*, and MCP-1 and high levels of iNOS expression, contributing to insulin resistance in different tissues [71–73]. 

When insulin binds to its receptor, IRS-1 and IRS-2 (insulin-receptor substrates 1 and 2) are recruited to its cytoplasmic region, which permits the binding and activation of two important kinases, the first of which is PI3K (phosphatidylinositol 3-kinase), and the second of which is AKT (a protein kinase B) [74]. Once activated, these kinases can regulate glucose and lipid metabolism. However, activated JNK can induce the phosphorylation of serine residues on IRS-1/2, inhibiting their ability to couple to PI3K and thereby promoting insulin resistance. In fact, the expression of JNK and NF-*κ*B is increased in diabetic patients [73], suggesting an important role for these molecules in diabetes. In myeloid-specific I*κκ*-*β* (an activator of NF-*κ*B)-deficient mice, a decrease in proinflammatory cytokine production (IL-1*β*, IL-6, TNF-*α*, and MCP-1) and the inhibition of NF-*κ*B activation has been reported, avoiding, in this way, the development of insulin resistance [75]. 

CAM*φ*s have been confirmed to be directly involved in diabetes because it has been found that 30% of the transcripts expressed in the adipose tissue of HFD-treated mice encode characteristic macrophage proteins associated with this subpopulation [76]. Also, the expression of transcripts for MIP-1*α*, MCP-1, MAC-1, F4/80, and CD68 was associated with insulin subproduction and TNF-*α* release [77]. In addition, macrophage polarization to CAM*φ*s had a direct relationship with the development of lipid droplets [78]. These characteristics relate the activation of CAM*φ*s to the promotion of AT accumulation and insulin resistance. 

Interestingly, a macrophage phenotypic switch has been reported in the AT of HFD-treated mice compared with normal diet-treated mice. Lumeng et al., 2007 [4], reported the presence of a natural AAM*φ* population within the AT of lean mice, and interestingly, the phenotype of these cells shift to CAM*φ*s when the mice were HFD-treated. The authors also showed that the IL-10 produced by AAM*φ*s had the ability to block the pathological effects of TNF-*α* in adipose tissue during insulin sensitivity [4, 78, 79], suggesting that while CAM*φ*s have insulin resistance-inducing effects, AAM*φ*s have a protector role within AT. Recently, another inflammatory chemokine has been shown to be involved in the resistance to insulin and T2D. A-ZIP transgenic mice (these animals are insulin-resistant and hyperlipidemic), which have a deficiency in MCP-1, displayed decreased hyperglycemia, hyperinsulinemia, and hepatomegaly; moreover, these mice had increased levels of markers for AAM*φ*s, such as *Arg1* and *Chi313* [80]. 

Also of note, PPARs are ligand-dependent transcription factors that have important functions in FA transport, synthesis, storage, mobilization, activation, and oxidation. Three distinct types of PPARs have been characterized: PPAR*α*, PPAR*δ*, and PPAR*γ*. PPAR*α* and PPAR*δ* are involved in the oxidation of FFA, while PPAR*γ* contributes to adipogenesis and the storage of FA. PPAR*γ* expression is induced in M*φ*s by IL-4/IL-13 [81–83]. Recent reports have shown that PPAR*γ* is required for AAM*φ*s induction and maturation, and the absence of this molecule enhances obesity and insulin resistance in HFD mice [81]. Moreover, PPAR*δ*-deficient Kupffer cells cannot be alternatively activated, predisposing mice to develop hepatic steatosis and insulin resistance [84]. As mentioned above, AAM*φ* development is dependent on IL-4/IL-13 stimulation, which activates the transcription factor STAT-6. STAT-6-deficient mice are more prone to obesity, and oxidative stress in their AT makes them more susceptible to T2D development, which, in turn, is associated with the absence of AAM*φ*s [85]. 

The role of other cells in the regulation of insulin sensitivity is recognized principally because of evidence in experimental models. Eosinophil-deficient mice have a smaller AT-AAM*φ* population and gain more weight, which indicates that eosinophils are an important source of IL-4 in adipose tissue [15]. Likewise, *Nippostrongylus brasiliensis* infection induced the recruitment of eosinophils and AAM*φ*s, which promoted a strong Th2 response and decreased obesity and insulin resistance [15], suggesting that eosinophils contribute to AAM*φ* induction and prevent T2D. 

Collectively, these findings suggest that adipose tissue is an important source of inflammatory molecules during obesity and can induce insulin resistance due to the increased recruitment of CAM*φ*s, which, in turn, can amplify the inflammatory response, promoting development of T2D, while high numbers of AAM*φ*s in the adipose tissue have been involved in glucose tolerance and diabetes prevention (Figure 2).

## 3. Conclusions

There is no doubt that the incidence of diabetes has increased in recent years, perhaps reflecting changes in lifestyle with regard to diet and/or hygiene. One explanation for the increased incidence of T1D is the hygiene hypothesis, which suggests that low or null exposure to parasites, especially helminths or their antigens, promotes the development of autoreactive leukocytes that attack *β* cells, initiating the disease. Helminth infections in mice with T1D have proved to prevent the inflammatory cascade through a mechanism associated with AAM*φ* induction. AAM*φ*s have been implicated in the regulation of other autoimmune diseases, such as experimental autoimmune encephalomyelitis [86] and autoimmune colitis, suggesting that AAM*φ*s have a strong immunoregulatory role in the induction of autoantigen tolerance [87]. Therefore, it is likely that these cells are the main players in the regulation of T1D.

The importance of AAM*φ*s extends beyond the regulation of autoimmunity, which we reviewed in this paper. AAM*φ*s can also inhibit the development of T2D, mainly by reducing obesity and insulin resistance, two major etiological factors in the induction of this disease, while CAM*φ*s are associated with increasing inflammation, obesity, and insulin resistance. Interestingly, the use of helminth parasites to induce AAM*φ*s has proved to be effective in disease treatment by reducing hyperglycemia, obesity, and the incidence of T2D. 

Finally, while CAM*φ*s have a major role in the injury and inflammatory response in diabetes, AAM*φ*s appear to reduce inflammation during type 1 and type 2 diabetes, suggesting that these macrophage populations may be therapeutic targets. Thus, based on the results of the various reports reviewed in this paper, we can highlight the possible therapeutic use of diverse immune-modulatory molecules to counteract or negatively influence specific inflammatory and cytotoxic T-cell-activating properties of macrophages.

## Figures and Tables

**Table 1:** Helminths that reduce types 1 and 2 diabetes.

Helminths	Disease/model	Infection/antigen	Effect	Reference
*Taenia crassiceps *	T1D/MLD-STZ	Inf	Increased Th2 response, AAM*φ* induction, and decreased TNF-*α*, therefore less hyperglycemia and no insulitis	[7]
*Schistosoma mansoni *	T1D/NOD/MLD-STZ	Inf/Ag	Increased anti-inflammatory cytokines, such as IL-4, IL-10, IL-5, and IL-13, as a result, loop of Th2 response; Treg, eosinophil, and AAM*φ* generation	[8–11]
*Heligmosomoides polygyrus *	T1D/NOD	Inf	Th2 response induction; IL-4, IL-13, and IL-10 augmentation; AAM*φ*s in pancreatic and peripheral lymph nodes; inflammation and insulitis reduced; no Treg generation	[12]
*Litomosoides sigmodontis *	T1D/NOD	Inf/Ag	High IL-4 and IL-5; AAM*φ* and Treg induction; reduced inflammation and glycemia	[13]
*Trichinella spiralis *	T1D/NOD	Inf/Ag	Amplification of Th2 response; less injury in pancreas and glycemia	[14]
*Nippostrongylus brasiliensis *	T2D/obese	Inf	Th2 response; recruitment of eosinophils and AAM*φ*s; decreased obesity and insulin resistance	[15]
